# Complete Digital Workflow for Evaluation of the Three‐Dimensional Monson's Sphere Using Digital Scanning

**DOI:** 10.1002/cre2.70063

**Published:** 2025-03-11

**Authors:** Cheng Wen, Huan Huan Wang, Huo Jia Muhetaer, Fan Xie, Rui Han, Jin Cheng Wu

**Affiliations:** ^1^ Department of Stomatology The Third Affiliated Hospital of Shenzhen University, Shenzhen Luohu Hospital Group Luohu People's Hospital Shenzhen Guangdong China

**Keywords:** Digital intraoral scan, Monson's sphere, Three‐dimensional models

## Abstract

**Background:**

As a combination of curve of Spee and curve of Wilson, the Monson sphere reflects the arrangement of teeth in three‐dimensional (3D) space. For occlusal analysis, the Monson sphere can provide an important reference for prosthetic reconstructions or orthodontic treatments.

**Objectives:**

The purpose of this study was to generate and investigate the 3‐D Monson sphere through a complete digital workflow with intraoral scanning models and custom software.

**Material and Methods:**

Sixty‐four Chinese adults (32 males and 32 females) with individual normal occlusion were recruited, and their intraoral scanning models were obtained using a digital intraoral scanner. Twenty‐six landmarks on the scanning models were digitized using a reverse engineering software. Monson's sphere was generated and evaluated by fitting a sphere to the cusp tips using a least‐squares method by means of custom software program. Mann–Whitney's *U* test was performed to test the radius difference of Monson's sphere between males and females. One‐sample *t*‐test was used to test the statistical significance (α = 0.05).

**Results:**

Monson's sphere could be generated by means of digital scanning combined with customed software. The mean radius of Monson's sphere was 77.35 ± 13.38 mm, which was lower than the theoretical value proposed by Monson. Furthermore, there were significant differences between males and females in Monson's sphere radius (*p* < 0.001).

**Conclusions:**

This study explores a new best‐fit algorithm for generating 3‐D Monson's sphere by complete digital workflow. The radius of Monson's sphere in Chinese adults was lower than the classical value of four‐inch proposed by Monson. There was a significant difference between males and females. Monson's sphere value found in this study could be used as a reference for prosthetic reconstruction and orthodontic treatment and be applied to improve dental treatment results.

## 1 Introduction

Teeth are not vertically arranged in the alveolar bone but exhibit a certain inclination direction and angle forming a suitable occlusion so that teeth on the upper and lower jaw are in close occlusal contact (Rengifo et al. 2019). The occlusion of teeth involves many aspects such as occlusal curve, occlusal plane, overlap and overlay of the anterior teeth, and the relationship of the canines and the molars. Appropriate occlusal curve and curvature can ensure that the mandible avoids occlusal interference and improves chewing performance during functional movement, which is of great significance for maintaining the stability of the dental arch and the functional health of the Stomatognathic System (Dritsas et al. 2021; Fueki, Yoshida, and Igarashi 2013; Shu et al. 2021). An abnormally arranged occlusal curve may lead to occlusal interference and temporomandibular disorders such as pain and abnormal snapping (Delgado‐Delgado et al. 2021; Manfredini, Lombardo, and Siciliani 2017; Ito et al. 1997). Therefore, it is necessary to do a detailed analysis of the patient's occlusal curve and pay attention to the curvature in the process of prosthetic reconstructions or orthodontic treatments.

The occlusal curve includes the curve of Spee and Wilson, as well as the Monson sphere. The curves of Spee and Wilson are simplified descriptions of the teeth arrangement characteristics in the sagittal and frontal planes, which reflect the arrangement characteristics of teeth in mesiodistal and buccolingual inclination, respectively (Spee et al. 1980; GH 1911). The curve of Spee has been reported to permit protrusive disocclusion of the posterior teeth through the combination of condylar guidance and anterior guidance, while the curve of Wilson also permits lateral mandibular excursions free from posterior interferences (Lynch and McConnell 2002; Craddock et al. 2005). Dental cusps in adult dentitions are theoretically described as spherical, with the occlusal surfaces of all teeth touching a part of the surface of a sphere, called the curve of Monson (Monson 1920). As a combination of the curve of Spee and Wilson, the Monson sphere reflects the arrangement of teeth in three‐dimensional manner and has been used as a vital reference for prosthodontic reconstruction of the posterior dentition (Lynch and McConnell 2002).

Determining the standard value of occlusal curvature is of great significance in the examination, diagnosis, and treatment of occlusal disharmony. Diverse approaches have been adopted to quantify these occlusal curvatures. Conventional quantitative investigations measure occlusal curvature directly on dental casts by means of rulers (Andrews 1972), analog calipers (Little 1975), digital calipers (Babu et al. 2017), and Broadrick occlusal plane analyzers (Kashinatha et al. 2012). Unluckily, previous measurements from dental casts fail to ensure the accuracy and repeatability of reference points. To deal with this problem, indirect measurements of occlusal curvature from two‐dimensional (2‐D) scan images were conducted and promoted the emergence of analysis methods through the mathematical calculations together with the aid of customed software (Li and Zhang 2006; Wu and Liu 2016). However, a true curve of Monson sphere is a virtual three‐dimensional structure unlike the conceptual diagrams which depict a sphere in the frame of 2‐D images. Measurements from 2‐D images cannot generate Monson's sphere, which requires 3‐D information including x, y, and z coordinate values, and reflect the effective 3‐D information of the true ones proposed by Monson (Monson 1920). Therefore, it is necessary to use 3‐D tools to comprehend the true Monson sphere.

Thanks to recent advances in computer‐aided design and computer‐aided manufacturing (CAD‐CAM) technology, digital intraoral scanning has been introduced into dental research since its accuracy in forming a 3‐D model recording bone and teeth structures with surrounding mucosa (Rasaie, Abduo, and Hashemi 2021; Tabesh et al. 2021). In combination with specialized 3‐D reconstruction software, researchers have discovered that the digital scanning models can ensure anatomical recognition in terms of accuracy and repeatability (de Paris Matos et al. 2021; Kihara et al. 2020).

Therefore, the objective of this study was to both generate 3‐D Monson's sphere and measure its occlusal curvatures by means of digital intraoral scanning models and specialized 3‐D processing software. The effect of gender on Monson's sphere was also investigated.

## 2 Materials and Methods

### 2.1 Participants Selection

This study protocol was approved by the ethical committee of Shenzhen Luohu Hospital Group Luohu People's Hospital, Shenzhen, China (protocol No. 2023‐LHQRMYY‐KYLL‐005). Sixty‐four subjects (32 males and 32 females) were recruited by dental practitioners in private practice. The inclusion criteria were good general health, complete permanent dentition excluding the third molars, absence of obvious wear of teeth, absence of cross or open bite, and no or slight crowding and/or spacing within 3 mm. The exclusion criteria were a systemic, infectious, or neoplastic disease, history of orthodontic or orthognathic treatment, severe periodontal disease or dental caries, history of temporomandibular disorders, and severe malocclusion or occlusal wear.

### 2.2 Digital Scanning Models

**Figure 1:**
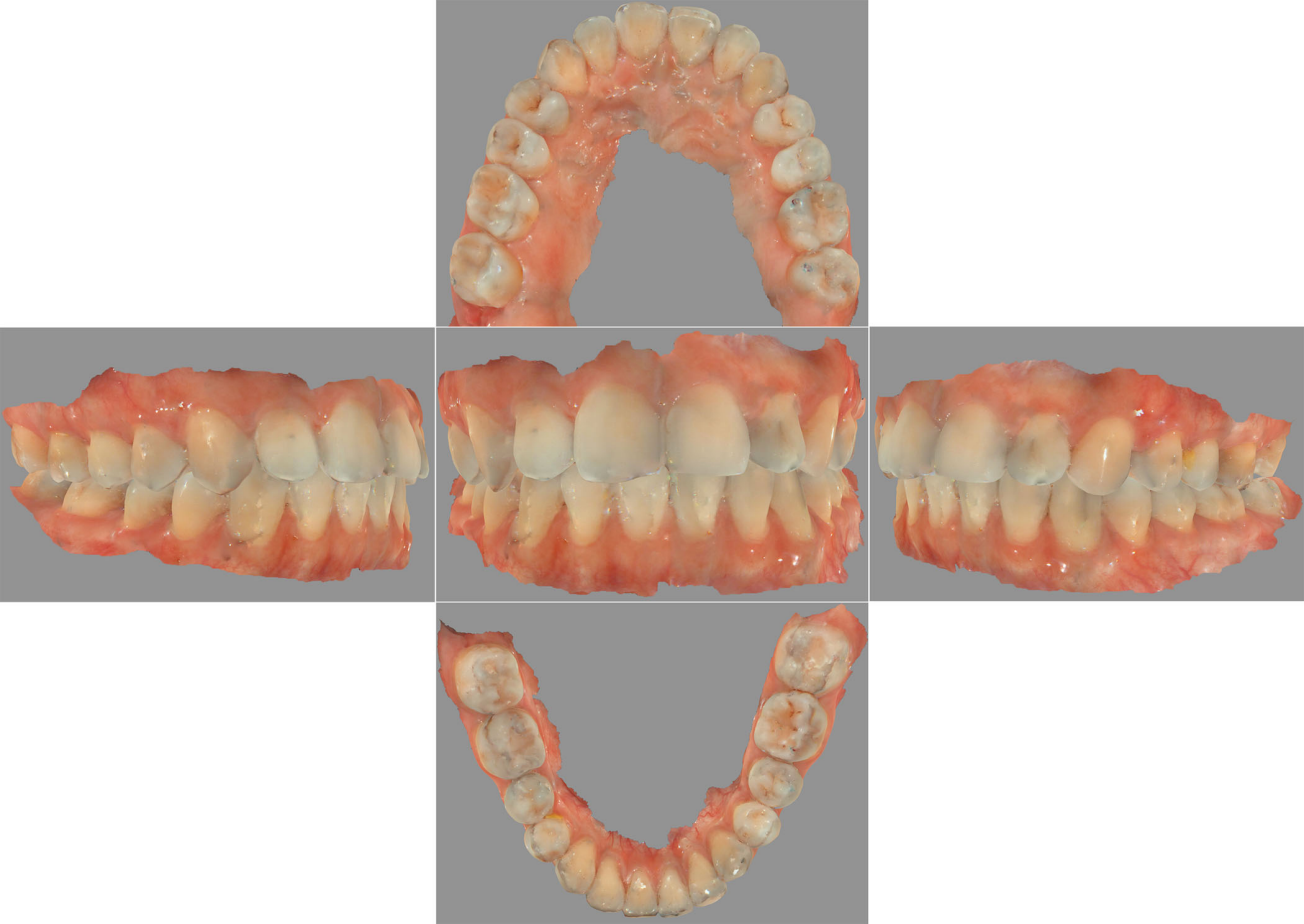
Digital intraoral scanning model. Different orientations of dentition.

Sixty‐four digital scanning models were captured and acquired from 32 males and 32 females with completely permanent dentition. In accordance with a scanning protocol based on the manufacturer's instructions, a digital intraoral scanner (Cerec AC, Sirona Dental Systems Inc, Bensheim, Germany) was applied to scan the whole dentition of the participants. In brief, scanning procedures followed the rule that the scanner tip not only began with the upper jaw and ended with the lower jaw but also started from the right side and continued to the left side along the arch all the way (Róth et al. 2020). The scanning sequence on the upper arch was occlusal then buccal and, eventually, palatal surface, whereas the occlusal, lingual, and buccal surfaces were scanned in order on the lower jaw. When scanning the occlusal surfaces, the scanner head was kept at 0–5 mm away from the tooth. For the scanning of the buccal and lingual surfaces, the scanner tip was rolled 45°–90° to the buccal and lingual sides, respectively (Yamamoto, Kataoka, and Manabe 2017). The high‐quality videos and images could be obviously viewed on a laptop screen throughout the scanning process, which allowed intuitive graphical feedback to identify the loss of scanning information. After scanning, the visual digital scanning models could be obtained (Figure 1); meanwhile, all acquired 3‐D videos and images were reprocessed as a stereolithography (STL) file, which would be subsequently used for the 3‐D analysis of reference points.

### 2.3 3D Location of Reference Points

**Figure 2:**
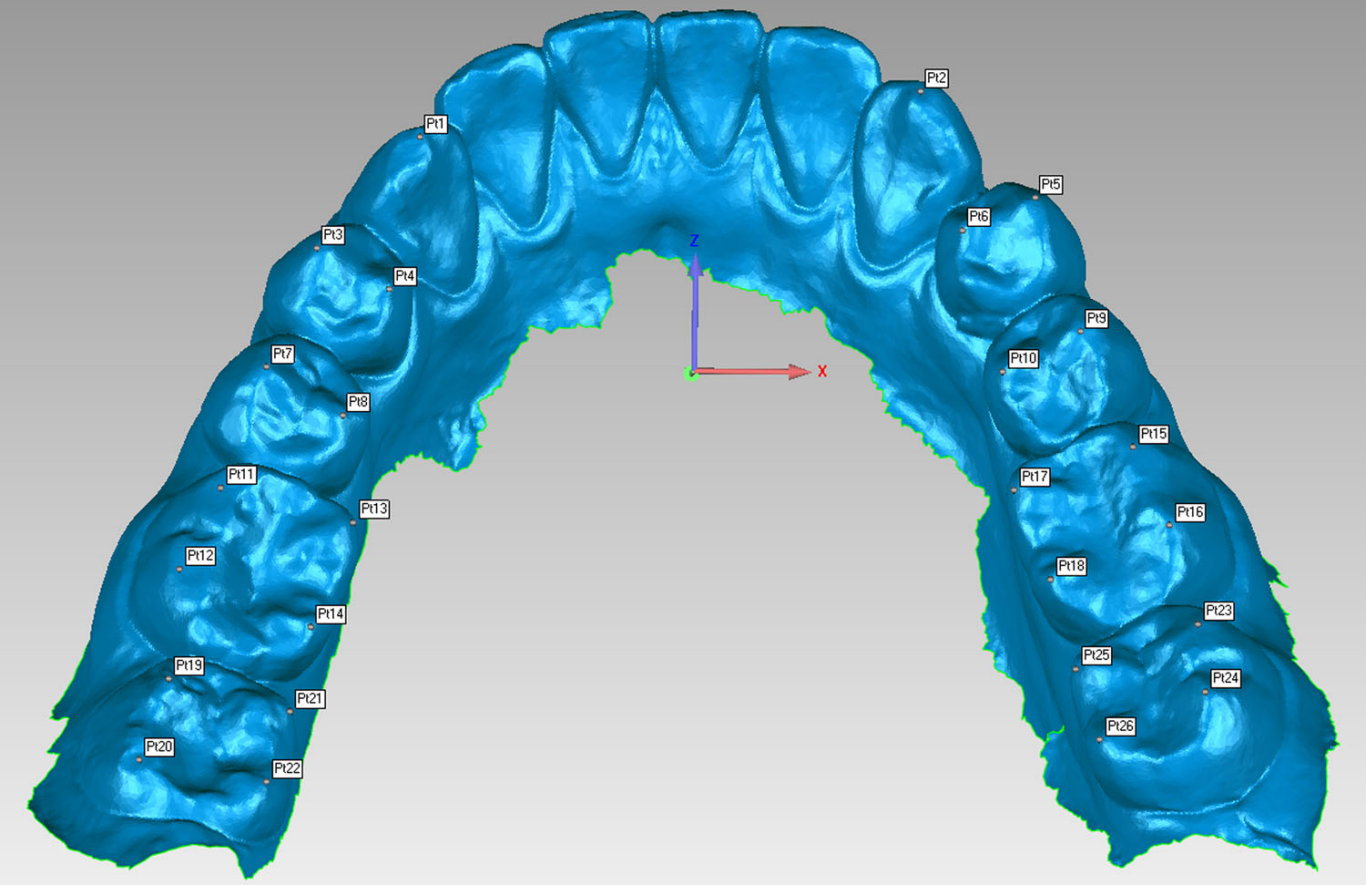
Digital reference points generation. About 26 reference points were identified. Cusp tips (Pt1, Pt2) were marked on canines. Buccal cusp tips (Pt3, Pt7, Pt5, Pt9) and lingual cusp tips (Pt4, Pt8, Pt6, Pt10) were marked on premolars. Mesio‐buccal cusp tips (Pt11, Pt15, Pt19, Pt23), disto‐buccal cusp tips (Pt12, Pt16, Pt20, Pt24), mesio‐lingual cusp tips (Pt13, Pt17, Pt21, Pt25), and disto‐lingual cusp tips (Pt14, Pt18, Pt22, Pt26) were marked on molars.

**Figure 3:**
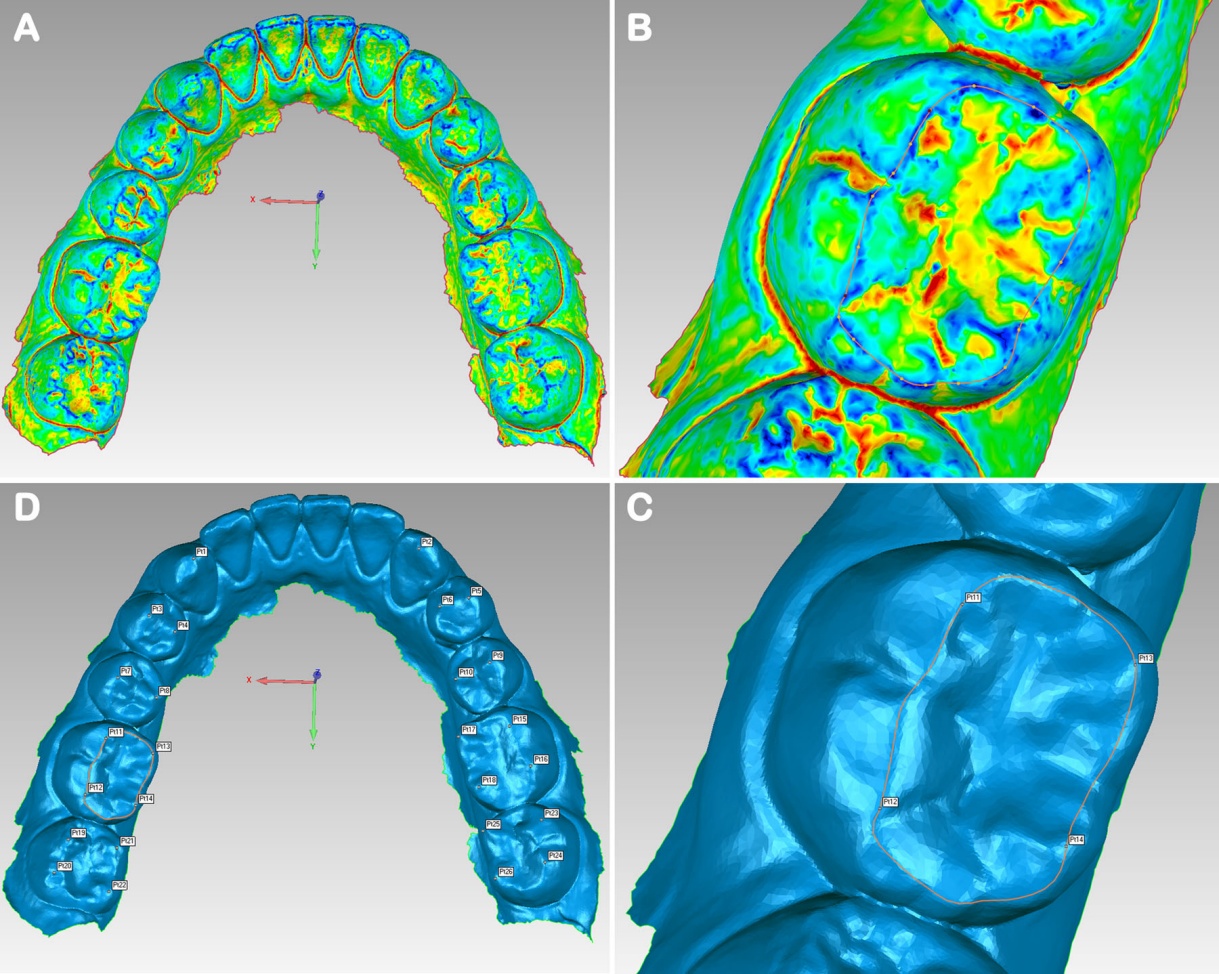
3‐D location of reference points. (A) Dentition was marked by the region of interest (red and yellow: pit and fissure, green: teeth tooth surfaces, blue: vertex height of teeth). (B) All the vertex height of teeth was circled through recognition of blue region. (C) Cusp tip was located by recognizing the deeper blue region of interest. (D) All the 26 reference points were marked.

In the present study, 26 reference points were generated to fit the Monson sphere, starting from the canine cusp tip, then continuing to the buccal cusp tips and finally to the lingual cusp tips of the premolar and molar teeth in order on the right and left sides (Figure 2). All acquired scanning models STL files were imported into reverse‐engineering software (Geomagic Studio 2013, Geomagic, Morrisville, USA). In the software, the digital scanning model can be freely rotated, zoomed in, and zoomed out to accurately locate reference points from a 3‐D perspective. The operator first converted the dentition model into the region of interest (Figure 3A). All the vertex height of teeth was circled through recognition of blue region (Figure 3B). Then, cusp tip was recognized by the deeper blue region of interest (Figure 3C). Finally, the operator clicked the “Analysis” button in the menu bar and then the “Point Coordinates” button and located the fitting reference points by clicking directly on the deeper blue region of interest (Figure 3D). After finishing 3‐D location of reference points, the generated coordinate (X, Y, Z) values of the corresponding reference points were recorded and exported into Excel files before fitting Monson's sphere.

### 2.4 Monson's Sphere Fitting

**Figure 4:**
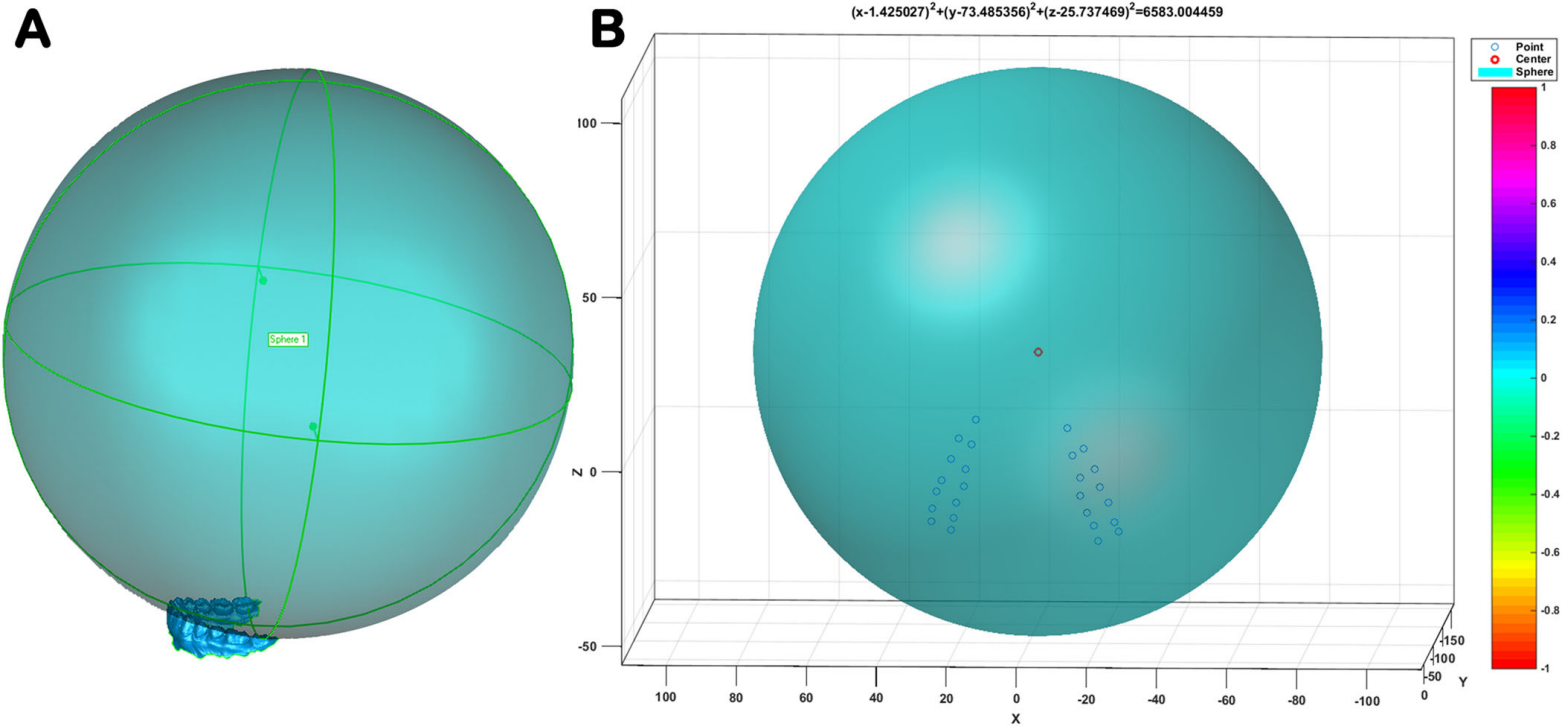
Monson's sphere fitting. (A) Monson's sphere with all the 26 reference points touching the sphere. (B) Sphere center and radius were obtained based on least squares method.

All the Excel files containing 26 coordinates (X, Y, Z) values were imported into Matlab R2014b software (Mathworks Inc., Natick, USA). A computation program (Appendix 1) was written to calculate and fit Monson's sphere based on the 3‐D coordinates values of 26 fitting reference points according to the least‐squares algorithm. Once running the calculation program, a perfectly fitted Monson's sphere was generated (Figure 4A), and the corresponding center coordinates values and the radius were obtained (Figure 4B).

### 2.5 Reproducibility of Measurement

The process of the reference point location and Monson's sphere fitting were performed by the same operator. In strict accordance with the previously stated requirements, 15 digital scanning models were selected at random and the reference point was marked twice on the models to obtain their corresponding coordinates (X1, Y1, Z1) and (X2, Y2, Z2) values. Subsequently, coordinate values were imported into Matlab R2014b software (Mathworks Inc., Natick, USA) to fit Monson's sphere. Finally, the radii of both R1 and R2 were recorded and measured using the intraclass correlation coefficient (ICC). As shown in Table 1, the ICC for intrarater reliability was 0.996 (95% confidence interval, 0.987–0.998, *p* < 0.001), indicating excellent repeatability of measurements.

**Table 1:** Intraclass correlation coefficient for intrarater reliability.

R1	R2
71.421	70.571
77.826	77.585
72.482	73.004
75.685	75.898
88.282	87.047
76.537	75.919
76.635	76.325
100.793	100.773
115.798	115.992
71.838	70.615
70.570	73.120
66.758	66.981
72.310	69.706
84.234	84.346
99.077	96.170

*Note:* ICC = 0.996, 95% confidence interval, 0.987–0.998, *p* < 0.001.

### 2.6 Statistical Analysis

The radius of Monson's sphere was expressed as mean ± standard deviation using conventional descriptive statistics. One‐sample *t*‐test was applied to analyze differences between samples and theoretical value, while Mann–Whitney's *U* tests were performed to examine differences in occlusal curvatures between males and females. For all analyses, *p* < 0.05 was considered significant.

## 3 Results

**Figure 5:**
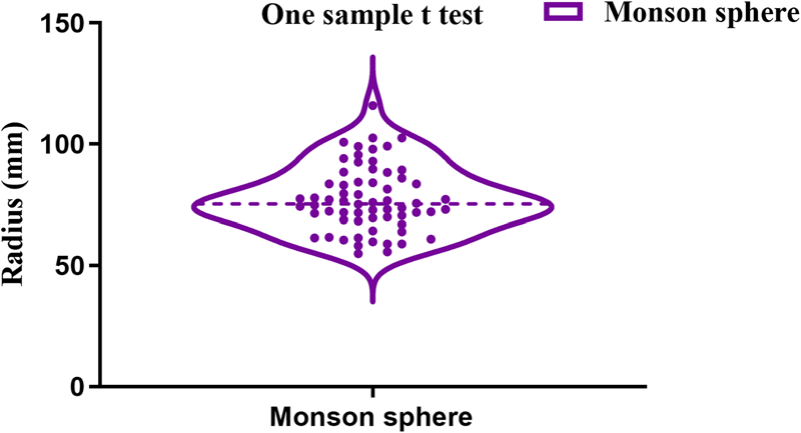
Comparison of Monson's sphere between samples and theoretical value. The radius of Monson's sphere for samples was smaller than that suggested by Monson (One sample *t* test, ****p < 0.0001).

**Figure 6:**
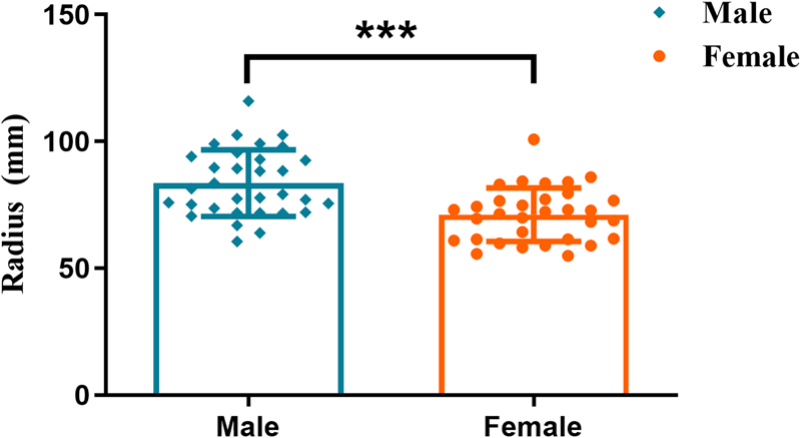
Comparison of Monson's sphere between males and females. The radius of Monson's sphere for females was smaller than that of the males (Mann–Whitney's *U* test, ***p < 0.001).

Descriptive statistics of variables studied are presented in Table 2. The total mean radius of Monson's sphere was 77.35 ± 13.38 mm (median, 75.36 mm; lower 95% CI of mean, 74.00 mm; upper 95% CI of mean, 80.69 mm) and was therefore lower than the original four‐inch value described by Monson (Figure 5, *p* < 0.0001). In addition, the mean radius of Monson's sphere for males and females was 83.57 ± 13.12 mm and 71.13 ± 10.59 mm, respectively. There were significant sex differences in Monson's sphere between males and females (Figure 6, *p* < 0.001).

**Table 2:** Sex differences in the radius of Monson's sphere.

Samples	Radius (mm) in Monson's sphere			
	Mean ± SD	Median	Lower 95% CI of mean	Upper 95% CI of mean
Males (*n* = 32)	83.57 ± 13.12	80.35	78.84	88.29
Females (*n* = 32)	71.13 ± 10.59	71.87	67.31	74.95
Total (*n* = 64)	77.35 ± 13.38	75.36	74.00	80.69

*Note:* Differences between males and females were determined by the Mann–Whitney *U* test.

## 4 Discussion

The development of human adult occlusion traverses three stages including primary dentition, mixed dentition, and permanent dentition, which take not less than 16 or 20 years to be established during this long process (Osborn and Francis 1989). In the case of occlusal disorder, two occlusal curvatures and an associated fitted sphere have been considered to reconstruct a functional occlusion (Nam et al. 2013). However, dentists prefer to utilize the curve of Spee and Wilson as the gold standard for occlusal reconstruction and fail to take Monson's sphere into account. Actually, the Monson sphere reflects the arrangement of teeth in three‐dimensional space and provides important reference for teeth arrangement devices (Bae, Park, and Kim 2021), articulators (Lepidi et al. 2021), and occlusal plane analyzers (Balavadivel et al. 2020).

Different from the curve of Spee and the curve of Wilson, the conceptual diagrams, which can be depicted and measured in the frame of 2‐D images, a real Monson sphere is not a 2‐D circle but a virtual 3‐D sphere which is challenging to produce due to the methodological limitation. In this study, a 3‐D Monson's sphere could be produced based on a novel fitting algorithm using digital scanning models (Figure 4). Published studies have explored many approaches in terms of analyzing the occlusal curvature of human dentition, whereas rare studies have been conducted to estimate the 3‐D morphology of occlusal surfaces. Ferrario and his coworker (Ferrario, Sforza, and Miani 1997) first analyzed the occlusal curvature by means of converting the 3‐D coordinates into 2‐D coordinates using a 3‐D digitizer. Subsequently, it is reported that a rough sphere, whose occlusal curvature was estimated by digitizing the radius and center position by the Broadrick occlusal analyzer (Kagaya et al. 2009), could be generated via an improved method with minor alterations on the basis of Ferrario's (Ferrario, Sforza, and Miani 1997). Notwithstanding that 3‐D coordinate reference points could form a rough sphere, the center of the sphere in these investigations was assumed on the y–z plane along with the x‐axis set at zero, which cannot accurately analyze the inherent 3‐D features of occlusal curvatures and produce a best‐fit sphere. Recently, researchers discovered that a 3‐D scanner could be applied to obtain the virtual dental models, on which reference points were determined using a custom software program (Nam et al. 2013). Although various examples of fitted spheres were generated, the best‐fitted spheres might be hard to find under the condition of no constraints. Hence, this study utilized digital scanning models together with a novel fitting method to directly generate a best‐fit Monson sphere to conquer the aforementioned shortcoming.

Precise positioning of reference points is the most crucial step in the process of fitting the Monson sphere. To ensure the reproducibility of measurement, a reverse engineering software was used to automatically identify the highest points of the cusp tip according to the region of interest in this study, which was similar to a recent study (Nam et al. 2013). In addition, the operator was admitted to freely rotate, zoom in, and zoom out the scanning models from a 3‐D perspective in the software, which could reduce the deviation of reference points. Eventually, the ICC test was performed to verify the repeatability of measurements, and the ICC for intra‐rater reliability was higher than 0.9 (Table 1), meaning high reliability for the measurements (Atenafu et al. 2012; Meseguer‐Henarejos et al. 2018).

In the course of occlusal reconstruction, how to accurately determine the occlusal plane is of great importance after dentists restore the occlusal vertical dimension (OVD) of patient (Goldstein, Goodacre, and MacGregor 2021; Shen et al. 2021). Clinically, dentists are accustomed to applying the original value proposed by Monson as a rough standard to figure out the occlusal plane. However, the application of an identical occlusal curvature may not be suitable for the physiological occlusion, resulting in occlusal interference and masticatory function disorder, since each patient is individually different (Surendran et al. 2016). Thus, it is indispensable to explore an applicable occlusal curvature for individual treatment. The total mean radius of the Monson sphere (77.35 ± 13.38 mm, Figure 5) measured in this study was smaller than European young adults data (male: 104.56 ± 17.96 mm, female: 99.7 ± 26.74 mm) (Ferrario, Sforza, and Miani 1997), which was close to the classical four‐inch value advocated by Monson (Monson 1920). In comparison with this study, another investigation revealed that occlusal curvature in South American young adults exhibited similar results (mean radius: 77.43 ± 17.55 mm) (Carneiro et al. 2024). On the contrary, Nam et al. (2013) obtained a larger mean radius (110.89 ± 25.75 mm) of Monson's sphere in Korean populations using a virtual 3‐D dental model. These distinct research findings could be interpreted by the differences of both race and methodology.

Apart from race and methodology, gender is considered as another pivotal factor that may affect the measurement of occlusal curvature (Surendran et al. 2016). In our case, as shown in Figure 6, there was a statistical difference in males' radius of Monson's sphere compared to females', with a mean difference of 12.44 mm (Table 2, *p* < 0.001). At present, the influence of sex on the occlusal curvature of Monson's sphere is still debatable. As for research involving two‐dimensional occlusal curve, it was concluded that sex differences were not connected to the occlusal curvature of the Spee curve (Halimi et al. 2018; Cheon et al. 2008). In findings similar to our results, Kagaya et al. (2009) and Nam et al. (2013) discovered that the radius of Monson sphere in women was significantly smaller than men's, indicating the sex difference in Monson's sphere radius.

To our knowledge, the four‐inch sphere theory proposed by Monson was founded on normal occlusion, the so‐called Angel Class I malocclusion. As a matter of fact, there are certain differences in occlusal curvature among patients with different types of malocclusion (Sayar and Oktay 2018). As for patients with Class II malocclusion, a smaller radius than four‐inch was recommended to relieve the steep anterior guidance and avoid the occurrence of posterior interferences (Babu et al. 2017). Conversely, Class III patients were prone to forming a steep curve in the posterior region, so that a larger occlusal curvature, typically a five‐inch radius, was suggested to flat the posterior curve (Lynch and McConnell 2002).

Indeed, occlusal curvature is variable since occlusal morphology of individuals changes with the occurrence of tooth wear over a lifetime. Occlusal wear is an irreversible process and, to some extent, is part of the physiological aging of permanent dentition (Peter et al. 2016). The processes that are involved are commonly classified as attrition (loss of mineralized tooth substance caused by tooth‐to‐tooth contact), abrasion (physical loss of mineralized tooth substance caused by objects other than teeth), and erosion (chemical loss of mineralized tooth substance caused by the exposure to acids) (Nadine et al. 2019). The excessive progression rate of wear both challenges the viability of teeth and leads to aesthetic and functional limitations, which is considered pathological (Berry and Poole 1976; Roos et al. 2023; David and Saoirse 2020). Moderate‐to‐severe occlusal wear (attrition) can result in reduction in tooth height that manifests as facets or cuppings (Berry and Poole 1976). It is unclear whether the occlusal curvatures could be applied to the worn dentition, but dentures in rehabilitation cases have been set up along the curve of Monson in the transverse plane to allow for movement along the sphere (Sengupta et al. 1999). Osborn found that the brunt of the wear occurred on the buccal cusp of the lower teeth and the palatal cusp of the upper teeth, which had an impact on occlusal curvatures of the Monson sphere (Osborn 1982). On account of this, our study restricted the inclusion criteria to minimize the effect of tooth wear on the occlusal curvature of the Monson sphere. Actually, during the clinical rehabilitation, the clinician should be aware of how much amount of occlusal curvature for the Monson sphere has to be restored so as to obtain stable occlusion.

In summary, the data found in this study may provide insight into the magnitude of the Monson sphere and improve treatment results in case of occlusal reconstructions. Nevertheless, when constructing an individual Monson's sphere matching the personalized dentition, various factors should be considered to precisely determine the inclination and direction of teeth. At this time, it is vague which factor exerts the foremost impact on the radius of the Monson sphere, and future research is necessitous to confirm the dominant factors that influence the radius such as gender, ethnicity, and malocclusion or other measurement methods.

## 5 Conclusions

Based on the outcome of this study, this study indicated that a novel fitting method could be applied to generate Monson's sphere by means of a complete digital workflow. The occlusal curvatures of Monson's sphere in Chinese subjects were lower than the theoretical four‐inch value proposed by Monson. There were significant differences between males and females in the radius of Monson's sphere, indicating that clinicians may take gender into consideration in terms of restoring occlusal function. The occlusal curvatures of Monson's sphere obtained in the present study may provide a significant guideline for occlusal reconstructions.

## Author Contributions

Jin Cheng Wu contributed to the conception and design of the study. Cheng Wen performed most of the experiments. Huan Huan Wang, Huo Jia Muhetaer, Fan Xie, and Rui Han performed the data analysis and prepared figures and tables. All authors participated in the drafting of the manuscript and the critical revision of the draft.

## Ethics Statement

This study was approved by the Ethics Committee of Shenzhen Luohu Hospital Group Luohu People's Hospital (approval number: 2023‐LHQRMYY‐KYLL‐005).

## Consent

All participants provided written informed consent.

## Conflicts of Interest

The authors declare no conflicts of interest.

## Supporting information

Supporting information.

## Data Availability

The data sets used and/or analyzed during the current study are available from the corresponding author on reasonable request.
