# Supplementary material for: Complete Digital Workflow for Evaluation of the Three‐Dimensional Monson's Sphere Using Digital Scanning
Source: Clin Exp Dent Res. 2025 Mar 11;11(1):e70063. doi: 10.1002/cre2.70063 (PMC11894269; doi:10.1002/cre2.70063)
Supplement: Supplementary file 1 — Supporting information. [file CRE2-11-e70063-s001.doc]

**The Fitting program for Monson sphere**

The sphere fitting process based on least-squares theory. The following fitting program was conducted to generate a best-fitting Monson sphere using Matlab R2014b software (Mathworks Inc., Natick, USA).

>> close all;

clear all;

sphere=load('C:\Users\Administrator\Desktop\NO.mat');

sphere=sphere.NO;

x=sphere(:,1);

y=sphere(:,2);

z=sphere(:,3);

data=[x,y,z];

f=@(p,data)(data(:,1)-p(1)).^2+(data(:,2)-p(2)).^2+(data(:,3)-p(3)).^2-p(4)^2;

p=nlinfit(data,zeros(size(data,1),1),f,[0 0 0 1]');% fitting parameters

r=p(4);

x0=p(1);

y0=p(2);

z0=p(3);

hold on

plot3(data(:,1),data(:,2),data(:,3),'o')

center=plot3(p(1),p(2),p(3),'o');

center.Color='red';

center.LineWidth=2;

[X,Y,Z]=meshgrid(linspace(-160,140));

V=(X-p(1)).^2+(Y-p(2)).^2+(Z-p(3)).^2-p(4)^2;

isosurface(X,Y,Z,V,0);

alpha .5;

camlight;

axis equal;

grid on;

view(40,40);

title(sprintf('(x-%f)^2+(y-%f)^2+(z-%f)^2=%f',p(1),p(2),p(3),p(4)^2));
